# F125. POOR OUTCOME SCHIZOPHRENIA (KRAEPELINIAN SUB-TYPE): SOCIAL COGNITIVE AND NEURODEVELOPMENTAL MARKERS

**DOI:** 10.1093/schbul/sby017.656

**Published:** 2018-04-01

**Authors:** Emmanuel Chevallier, Audrey Tanguy, Marie-Odile Krebs, Serge Mitelman, Monte Buchsbaum, Marie-Cécile Bralet

**Affiliations:** 1 FJ6 CHI Clermont de l’Oise; 2 AP-HP Paris; 3 SHU Ste Anne, Paris; 4 Icahn School of Medicine; 5 NeuroPet Center, UCSD; 6 Unité CRISALID CHI Clermont de l’Oise

## Abstract

**Background:**

Poor outcome schizophrenia represents a public health challenge and it asks questions about neurodevelopmental mechanisms by its own. The kraepelinian schizophrenia sub-type, defined by Keefe’s criteria (1987), refers to a very poor prognosis sub-group (severe dysfunction in self-care) on the basis of the longitudinal course of the illness. Studies on kraepelinian sub-group show differences with good outcome patients regarding pre-morbid functioning, negative and disorganized symptoms, impaired performance on specific social cognitive and motor deficits (visual-motor processing, abstraction/flexibility, fine motor dexterity) (Albus and al., 1996; Bralet and al., 2006). Neurological soft signs (NSS) refer to subtle neurological abnormalities comprising deficits in sensory integration, motor coordination and sequencing of complex motor acts. Previous studies showed that NSS scores are correlated to schizophrenia, specifically among patients with poor premorbid functioning and with severe negative and disorganization symptoms. NSS could be a neurodevelopmental marker interesting to detect patients with a risk of poor prognosis. Deficits in theory of mind is correlated with disorganization and poor prognosis. The aim of our study was to explore the association between NSS, Theory of Mind and kraepelinian sub-type in order to understand better the etiopathogenic mechanisms underlying the kraepelinian sub-type.

**Methods:**

In 2016, we recruited 2 samples of 25 schizophrenic patients, kraepelinians and no-kraepelinians, matched on sex, ages (+/- 5 years) and duration of illness (+/- 5 years) from the psychiatric departments in Picardie area (France), according to DSM-IV-TR criteria and using Keefe’s criteria. Several socio-demographical, pharmacological, clinical, cognitive and NNS (with the 3 subscores, sensory integration, motor integration and motor coordination) (Krebs and al., 2000) were collected for each patient. To compare the 2 sub-groups we used bi-variate analysis and multivariate regression analysis (p< 0,05)

**Results:**

Results showed a worse significant NSS score among kraepelinian patients: total score, sensory integration, motor integration, motor coordination, p< 0,0001; there was no link with treatment (equ mg/day chlorpromazine). As well kraepelinians show worse significantly performance at the eye gaze test p<0,001. Multivariate analysis showed that kraepelinian sub-type is more explained significantly by eyes-test, motor integration and disorganization dimension

**Discussion:**

Poor prognosis schizophrenia refers to specific and complex neurodevelopmental mechanisms which could be markers of a poor outcome. We must confirm these results in a larger prospective cohort from UHR and first episode assessing some specific neurodevelopmental markers using NSS and cognitive assessment. As well some specific biological markers and genes implicating in neurodevelopment and glutamatergic system could be studied in these patients. Focusing on these specific markers could contribute to define innovating combinating therapeutic strategies (pharmacological, cognitive remediation and social skills) to avoid poor prognosis.

